# Mitogen-activated protein kinase phosphatase 1 controls broad spectrum disease resistance in *Arabidopsis thaliana* through diverse mechanisms of immune activation

**DOI:** 10.3389/fpls.2024.1374194

**Published:** 2024-03-21

**Authors:** Diego José Berlanga, Antonio Molina, Miguel Ángel Torres

**Affiliations:** ^1^ Centro de Biotecnología y Genómica de Plantas, Universidad Politécnica de Madrid (UPM) - Instituto Nacional de Investigación y Tecnología Agraria y Alimentaria (INIA/CSIC), Madrid, Spain; ^2^ Departamento de Biotecnología-Biología Vegetal, Escuela Técnica Superior de Ingeniería Agronómica, Alimentaría y de Biosistemas, UPM, Madrid, Spain; ^3^ Center of Excellence for Plant Environment Interactions (CEPEI), Madrid, Spain

**Keywords:** *Arabidopsis thaliana*, plant immunity, reactive oxygen species (ROS), MKP1, RBOHD, signaling pathways, necrotrophic fungi, *Pseudomonas syringae*

## Abstract

*Arabidopsis thaliana* Mitogen-activated protein Kinase Phosphatase 1 (MKP1) negatively balances production of reactive oxygen species (ROS) triggered by Microbe-Associated Molecular Patterns (MAMPs) through uncharacterized mechanisms. Accordingly, ROS production is enhanced in *mkp1* mutant after MAMP treatment. Moreover, *mkp1* plants show a constitutive activation of immune responses and enhanced disease resistance to pathogens with distinct colonization styles, like the bacterium *Pseudomonas syringae* pv. tomato DC3000, the oomycete *Hyaloperonospora arabidopsidis* Noco2 and the necrotrophic fungus *Plectosphaerella cucumerina* BMM. The molecular basis of this ROS production and broad-spectrum disease resistance controlled by MKP1 have not been determined. Here, we show that the enhanced ROS production in *mkp1* is not due to a direct interaction of MKP1 with the NADPH oxidase RBOHD, nor is it the result of the catalytic activity of MKP1 on RBHOD phosphorylation sites targeted by BOTRYTIS INDUCED KINASE 1 (BIK1) protein, a positive regulator of RBOHD-dependent ROS production. The analysis of *bik1 mkp1* double mutant phenotypes suggested that MKP1 and BIK1 targets are different. Additionally, we showed that phosphorylation residues stabilizing MKP1 are essential for its functionality in immunity. To further decipher the molecular basis of disease resistance responses controlled by MKP1, we generated combinatory lines of *mkp1-1* with plants impaired in defensive pathways required for disease resistance to pathogen: *cyp79B2 cyp79B3* double mutant defective in synthesis of tryptophan-derived metabolites, *NahG* transgenic plant that does not accumulate salicylic acid, *aba1-6* mutant impaired in abscisic acid (ABA) biosynthesis, and *abi1 abi2 hab1* triple mutant impaired in proteins described as ROS sensors and that is hypersensitive to ABA. The analysis of these lines revealed that the enhanced resistance displayed by *mkp1-1* is altered in distinct mutant combinations: *mkp1-1 cyp79B2 cyp79B3* fully blocked *mkp1-1* resistance to *P. cucumerina*, whereas *mkp1-1 NahG* displays partial susceptibility to *H. arabidopsidis*, and *mkp1-1 NahG*, *mkp1-1 aba1-6* and *mkp1-1 cyp79B2 cyp79B3* showed compromised resistance to *P. syringae*. These results suggest that MKP1 is a component of immune responses that does not directly interact with RBOHD but rather regulates the status of distinct defensive pathways required for disease resistance to pathogens with different lifestyles.

## 1 Introduction

Plant defense responses are orchestrated through complex signaling networks that involve both early and sustained response mechanisms, collectively contributing to the activation of different defense layers (Yuan et al., 2021). At the forefront of this defense arsenal are Pattern Recognition Receptors (PRRs) located at the plasma membrane of plant cells. These PRRs, mainly Receptor Kinases (RKs) and Receptor-Like Proteins (RLPs), sense the presence of invading pathogens in two ways: i) through the direct recognition of conserved molecules present in the pathogens, called Microbe-Associated Molecular Patterns (MAMPs; Bender and Zipfel, 2023); ii) through the recognition of plant derived-molecules, called Damage-Associated Molecular Patterns (DAMPs), released or synthesized after pathogen attack (De Lorenzo and Cervone, 2022). The engagement of PRRs initiates Pattern Triggered Immunity (PTI) responses that includes a cascade of events: apoplast alkalinization, cytoplasmic calcium influxes, reactive oxygen species (ROS) production by NADPH oxidases, phosphorylation cascades triggered by Mitogen-Activated Protein Kinases (MPKs) and Calcium Dependent protein Kinase (CPKs), and transcriptional reprogramming (DeFalco and Zipfel, 2021). The coordination of these signaling events leads to the synthesis of antimicrobial compounds, like antimicrobial peptides and metabolites (e.g. Tryptophan (Trp)-derived indol-glucosinolates), reinforcement of the cell wall, and other defense-related processes that collectively contribute to restrict pathogen colonization. Additionally, intracellular nucleotide-binding domain and leucine-rich repeat-containing receptors (NLRs) recognize pathogen derived effectors, further promoting the activation of these defenses through a different layer of disease resistance mechanism that is termed Effector-Triggered Immunity (ETI; Ngou et al., 2022).

Despite the necessity of robust activation of defense responses for effective plant disease resistance, there is a delicate control of the intensity and long-lasting of these responses to avoid an over activation of PTI/ETI. Induction of defense responses can compromise the normal development of the plant, impacting growth and reproduction (Huot et al., 2014). Excessive or prolonged activation of defense mechanisms can lead to resource allocation away from normal physiological processes, potentially hindering the plant’s ability to thrive (Monson et al., 2022). Therefore, it is essential to limit the induction of defenses to prevent detrimental effects on the overall fitness of the plant. Different mechanisms have been described to contribute to limit the induction of defensive response, like dephosphorylation of activated proteins by phosphatases, endocytosis of PRRs, or ubiquitination and degradation of activated proteins (Beck et al., 2012; Couto et al., 2016; Zhang and Zeng, 2020).

One critical aspect of the regulation of defense responses is the control of ROS production. ROS are produced during early PTI responses and are potentiated through ETI to a second wave with more sustained ROS accumulation (Castro et al., 2021; Wu et al., 2023). NADPH oxidases, called in plants RESPIRATORY BUST OXIDASE HOMOLOGS (RBOHs), are the main enzymes that account for most of these pathogen dependent ROS production (Torres and Dangl, 2005). They are plasma membrane proteins that transfer electrons from cytosolic NADPH to apoplastic oxygen, resulting in the production of superoxide (O_2_^-^), which rapidly dismutates to hydrogen peroxide (H_2_O_2_). H_2_O_2_, a more stable ROS, modulates downstream cellular targets, largely by oxidizing redox-active cysteines and other amino acids, and by travelling through the apoplast, spreading the stress signal to various regions of the plant (Bleau and Spoel, 2021; Castro et al., 2021). While ROS play a vital role in signaling, their overproduction can lead to oxidative damage, negatively impacting cellular structures and functions (Mittler, 2017). Therefore, plants possess a battery of antioxidant and detoxifying enzymes that limit ROS accumulation (Das and Roychoudhury, 2014). Moreover, ROS production is tightly controlled making its accumulation transitory, not exceeding the necessary threshold for ROS effective regulatory function and avoiding ROS excessive accumulation due to their potentially damaging effects.

In the model plant *Arabidopsis thaliana* the NADPH oxidase RBOHD is the key oxidase responsible for most pathogen-induced ROS production (Torres et al., 2002). The activation of this oxidase has been well characterized and involves multiple posttranslational modifications mainly acting at the cytosolic N-terminal domain of the protein, which contains Ca^2+^ binding EF hands (Castro et al., 2021). Upon pathogen recognition, several kinases act in concert at this N-terminal domain to regulate RBOHD activation (Kadota et al., 2015; Castro et al., 2021; Wu et al., 2023). These kinases that phosphorylate RBOHD N-terminal include: i) receptor-like cytoplasmic kinases (RLCKs) like BOTRYTIS INDUCED KINASE 1 (BIK1; Kadota et al., 2014), which plays a preeminent role in RBOHD regulation, and the RESISTANCE TO PSEUDOMONAS SYRINGAE PV. MACULICOLA 1-INDUCED PROTEIN KINASE (RIPK; Li et al., 2021); ii) RKs, like DOES NOT RESPOND TO NUCLEOTIDES 1, (DORN1; Wang et al., 2018); iii) MPKs like SERINE/THREONINE KINASE 1 (SIK1; Zhang et al., 2018); iv) and the CALCIUM-DEPENDENT PROTEIN KINASE 5 (CPK5; Dubiella et al., 2013). The precise orchestration of RBOHD activation involves convergent phosphorylation events at some specific RBOHD Ser/Tyr residues (e.g. Ser343 and Ser347) by these kinases, contributing to the fine-tuning of ROS production in response to different stimuli (Wu et al., 2023). Furthermore, phosphorylation of C-terminal residues by receptors like CYSTEINE-RICH RECEPTOR KINASE 2 (CRK2) and persulfidation of specific Cys in the C-terminus also contribute to the activation of AtRBOHD-dependent ROS production, emphasizing the complexity and versatility of regulatory mechanisms governing plant immunity mediated by RBOHD (Kimura et al., 2020; Shen et al., 2020).

Contrary to activation, fewer mechanisms are known to negatively regulate RBOHD-activity and its de-phosphorylation. Prior pathogen elicitation, transcriptional and translational control could limit RBOHD protein level to restrict ROS production (Morales et al., 2016; George et al., 2023). Also, the ubiquitination mechanism mediated by the RLCK AvrPphB SUSCEPTIBLE1-LIKE 13 (PBL13) contributes to maintain the appropriate RBOHD levels at the plasma membrane at the resting state (Lee et al., 2020). PBL13 phosphorylation of the C-term of the protein drives its ubiquitination (Lee et al., 2020), leading to RBOHD degradation in the vacuole with the contribution of XYLEM CYSTEINE PEPTIDASE 1 (XCP1; Liu et al., 2023). Once defense signaling is engaged, two mechanisms could contribute to deactivate the active RBOHD and prevent excessive ROS production. Over-accumulation of ROS in the cytosol is sensed by QUIESCIN SULFHYDRYL OXIDASE HOMOLOG 1 (QSOX1), which interacts with and oxidizes S-nitrosoglutathione reductase AtGSNOR, elevating intracellular S-nitrosoglutathione (GSNO) levels (Chae et al., 2021). High GSNO levels can promote S-nitrosylation of Cys890 in RBOHD, which inactivates the oxidase (Yun et al., 2011). Also, a recent work documents the interaction of PHAGOCYTOSIS OXIDASE/BEM1P (PB1) DOMAIN-CONTAINING PROTEIN (PB1CP) with RBOHD to negative regulates MAMP-induced ROS production. PB1CP could negatively regulate the active oxidase by competing for binding with activating kinases, such as BIK1, and by promoting endocytosis, which could lead to the degradation of the oxidase (Goto et al., 2023).

*Arabidopsis thaliana* Mitogen-activated protein Kinase Phosphatase 1 (MKP1) regulates various cellular processes, including growth, development, and stress responses (Ulm et al., 2002; Bartels et al., 2009; Tamnanloo et al., 2018). This phosphatase appears to function by dephosphorylating and inactivating MPKs, showing a strong interaction with MPK3 and MPK6 (Ulm et al., 2002; Bartels et al., 2009) that are two positive regulators of immune responses, like PTI (Ren et al., 2002). In the context of *Arabidopsis thaliana* immunity, MKP1 has emerged as an important negative regulator of PTI and disease resistance (Anderson et al., 2011; Escudero et al., 2019). Consequently, *Arabidopsis thaliana mkp1* mutant alleles display broad spectrum disease resistance, but also some detrimental chlorosis and necrosis in their leaves at later stages of plant development or under some stress conditions (Bartels et al., 2009; Jiang et al., 2017; Escudero et al., 2019). The mechanism explaining how MKP1 exerts these multifaced functions is unclear. MKP1 has been proposed to function as a repressor of salicylic acid (SA) synthesis and signaling (Bartels et al., 2009). We identified an *mkp1-2* allele in a mutant suppressors screening of the highly susceptible *agb1-2* plants, that are impaired in β subunit (AGB1) of the heterotrimeric G protein, which is a key regulator of immune responses in *Arabidopsis thaliana* (Trusov et al., 2010; Torres et al., 2013; Escudero et al., 2019). Notably, we found that MKP1 was a negative regulator of ROS production, since *mkp1* mutants displayed enhanced ROS accumulation in response to the MAMPs flg22 and chitin. These results highlighted MKP1 importance in fine-tuning the balance of ROS production and immune responses activation. Therefore, we aimed to investigate if MKP1 has a direct role in the inactivation of RBOHD during the immune response. Moreover, to further characterize the distinct facets of plant defense regulated by MKP1, we set to characterize genetically the contribution of different signaling pathways to the immune function of MKP1 during *Arabidopsis thaliana* disease resistance responses to pathogens with different lifestyles.

## 2 Methods and materials

### 2.1 Plant material and growth conditions

All *Arabidopsis thaliana* lines used were in Columbia-0 (Col-0) background. *mkp1-1* allele and line *NahG mkp1-1* were obtained by R. Ulm (Bartels et al., 2009). The allele *mkp1-2*, that has a weaker phenotype in disease resistance than *mkp1-1* allele, was described previously (Escudero et al, 2019). Lines expressing *RBOHD* under its own promoter (*pRBOHD*, abreviated *pD)*, *pD::FLAG::RBOHD*, *pD::FLAG::RBOHD^S39A/S339A/S343A^* and *pD::FLAG::RBOHD^S343/S347^* (all 4 in *rbohD* background) were obtained from Y. Kadota (Kadota et al., 2014, Kadota et al., 2019). Lines *35S::MYC::MKP1* and *35S::MYC::MKP1^4A^* (with the 4 putative regulatory phosphosites mutated to Ala) in *mkp1-1* background were obtained from S. Peck (Jiang et al., 2017). *bik1* mutant and *pBIK::BIK1::HA* line were provided by Cyril Zipfel (Kadota et al., 2014). Other lines used in this work were: *agb1-2* (Ullah et al., 2003), *mpk3-1* and *mpk6-2* (Beckers et al., 2009), *NahG* (Delaney et al., 1994), *cyp79B2 cyp79B3* (abbreviated in figures *cyp79B2/B3*), *aba1-6* and *abi1-2 abi2-2 hab1-1* (abbreviated *abi1/2 hab1*; Sánchez-Vallet et al., 2012).

Different transgenic lines and mutant combinations with *mkp1-1* and *mpk1-2* were generated by manual crosses and homozygous lines were identified by PCR. These include: *pD::FLAG::RBOHD rbohD mkp1-1, pD::FLAG::RBOHD^S39A/S339A/S343A^ rbohD mkp1-1, pD::FLAG::RBOHD^S343/S347^ rbohD mkp1-1, mkp1-2 bik1*, *mkp1-2 mpk3-1, mkp1-2 mpk6-2, mpk1-1 cyp79B2 cyp79B3, mkp1-1 aba1-6, mkp1-1 abi1/2 hab1-1, mkp1-1 NahG.*

For soil-based plant growth, *Arabidopsis thaliana* seeds were sown, stratified at 4°C for 3 days in darkness, and moved to a grown chamber at 22°C, 80% relative humidity, under short day photoperiod (10-h light/14-h dark) and light intensity of 110-120 µE/m^2^/s. For *in vitro* plant growth, sterilized seeds were sown in ½ strength Murashige and Skoog (MS) medium containing 1% sucrose and subsequently stratified for 3 days in the dark at 4°C. Seeds were germinated at 22°C, and grown in a plant growth chamber under long day photoperiod (14-h light/10-h dark) and a light intensity of 150 µE/m^2^/s.

### 2.2 ROS measurement

H_2_O_2_ production was determined by a luminol-based assay. Four mm diameter disc leaves from 4-5 week-old *Arabidopsis thaliana* plants (n = 8) were collected in 96-wells white plates (Thermo Scientific) and incubated overnight in 100 μl of ROS Buffer (100 μg/ml peroxidase, Sigma; and 100 nM Luminol, Sigma). Luminescence was measured as RLU (relative light units) every minute over 40 minutes after induction with 1 μM flg22 or 50 μM chitohexaose (CHI6) in a Varioskan LUX luminometer (Thermo Scientific), as described in Torres et al. (2013).

### 2.3 Disease resistance assays

For *Plectospharella cucumerina* BMM (*Pc*BMM) assays, 16-17-day-old plants were spray inoculated with a 4 × 10^6^ fungal spores/ml suspension. For each genotype, 3 tubes with 8-10 plants were collected 4-5 days-post-inoculation (dpi) for DNA genomic extraction. *Pc*BBM biomass was determined by qPCR, using specific primers FW 5-CAAGTACGTTCCCCGTGCCG-3 and RV 5-GAAGAGCTGGCCGAAGGGACC-3 for *Pc β-TUBULIN*. Samples were standardized against *AtUBIQUITIN* using specific primers FW 5- AAAGGACCTTCGGAGACTCCTTACG-3 and RV 5- GGTCAAGAATCGAACTTGAGGAGGTT-3. *agb1-2* plants were used as a hypersusceptible control (Escudero et al., 2019).

For *Pseudomonas syringae* pv. tomato (*Pto*) DC3000 resistance assays, 21-day-old plants were spray inoculated with a bacterial suspension at a concentration of 3 x 10^8^ colony forming units (cfu)/ml with 0.04% Silwet L-77. Four samples were collected per genotype at 4 dpi, each one containing 4 mm diameter discs. Material collected was ground and plated on Kings B media plates after serial dilution to count cfu. *agb1-2* plants served as a hypersusceptible control (Torres et al., 2013).

For *Hyaloperonospora arabidopsidis* (*Hpa*) Noco2 resistance assays, 10-11-day-old seedlings were spray inoculated with a 4 × 10^4^ spores/ml suspension. For each genotype, 3 tubes with 12-15 seedlings were collected 6 dpi for DNA genomic extraction. *Hpa* biomass was determined by qPCR, using specific primers FW 5-ATCTTCATCATGTAGTCGGTCAAGT-3 and RV 5-GTGTCGCACACTGTACCCATTTAT-3 for *Hpa ACTIN*. Samples were standardized against *AtUBIQUITIN*. *NahG* plants were used as a susceptible control (Delaney et al., 1994).

All these different pathology experiments were repeated at least three times with similar results.

### 2.4 Protein extraction, immunoprecipitation and immunodetection

Twelve-day-old *Arabidopsis thaliana* seedling (n = 15-20) growth *in vitro*, were treated with 500 nM flg22 or H_2_O for 10 minutes before fast-freezing in liquid nitrogen. Total proteins were manually ground in cold and incubated in extraction buffer [50 mM Tris-HCl pH 7.5, 200 mM NaCl, 10 mM NaF, 1 mM EDTA, 2 mM sodium orthovanadate, 1 mM sodium molybdate, 10% (v/v) glycerol, 0.1% (v/v) Tween-20, 1 mM 1,4-dithiothreitol, 1 mM phenylmethylsulfonyl fluoride and phosphatase inhibitor cocktail (Sigma-Aldrich)] for 1 hour at 4°C. Protein samples were quantified by a Bradford assay and subsequently normalized to a total protein concentration of 3-5 mg/ml.

For immunoprecipitation, samples were incubated with 20 µl of anti-MYC, anti-FLAG or anti-HA microbeads (µMACS, Miltenyi Biotec) for 2 hours. Proteins were then retained in µColumns and eluted in SDS loading buffer following Miltenyi Biotec instructions.

For co-immunoprecipitations and control loading, proteins were separated by electrophoresis using 4–15% Mini-PROTEAN TGX Gels (BIO-RAD) for 1 hour and 20 minutes at 120 V in running buffer (Laemmli). Proteins were transferred to nitrocellulose membranes using iBlot 3 Western Blot Transfer Device (Invitrogen). Membranes were blocked in TBST (Tris-buffered saline, 1% Tween-20) and 5% powder milk for 2 hours at room temperature, and subsequently incubated with anti-FLAG (from mouse; 1:2500 dilution; Merck Life Science S.L.U), anti-MYC (from mouse; 1:2500 dilution; Merck Life Science S.L.U.) or anti-HA (from rat; 1:5000 dilution; Milteny Biotec) antibodies in TBST with 3% milk at 4°C overnight. After three 10-minute washes with TBS, membranes were incubated with secondary antimouse-HRP (1:2500 dilution; Sigma Aldrich) or antirat-HRP (1:5000 dilution; Sigma Aldrich) antibodies in TBST with 3% powder milk for two hours at room temperature. After three 10-minute washes with TBS, proteins on the membranes were detected using ECL western blotting substrate (Thermo Fisher Scientific) and images were taken using iBright FL1000 Image System (Thermo Fisher Scientific). For loading controls, membranes were stained with Ponceau-S Red (Sigma Aldrich).

### 2.5 RNA extraction and quantification of gene expression

Collected plant material from uninfected and infected plants was fast-frozen using liquid nitrogen. After manual grinding, RNA extraction was performed using the RNeasy kit (QUIAGEN) including DNAse treatment following the supplier´s instructions. cDNA was generated using Transcriptor First Strand cDNA Synthesis kit (Roche Applied Science). For qRT-PCR analysis, reactions were performed with 40 ng of cDNA using SYBR green master mix system (Roche Applied Science). PCR conditions were as follows: 95°C for 10 min and then 45 cycles of 95°C for 15 seconds and 60°C for 1 minute. A dissociation stage was carried out at the end confirming only single products were generated. Primers used were as follows: for *PR1*: FW 5-CGTCTTTGTAGCTCTTGTAGGTGC-3 and RV 5-TGCCTGGTTGTGAACCCTTAG-3; for *PDF1-2*: FW 5-TTCTCTTTGCTGCTTTCGACG-3 and RV 5-GCATGCATTACTGTTTCCGCA-3; for *PAD3*: FW 5-CAACAACTCCACTCTTGCTCCC-3 and RV 5-CGACCCATCGCATAAACGTT-3; for *CYP81F2*: FW 5-TATTGTCCGCATGGTCACAGG-3 and RV 5-CCACTGTTGTCATTGATGTCCG-3. *UBC21* (*At5g25760*) expression (FW 5-GCTCTTATCAAAGGACCTTCGG and RV 5- CGAACTTGAGGAGGTTGCAAAG) was used for normalizing each gene expression level using the Pfaffl method (Pfaffl, 2001).

### 2.6 Statical analyses

Data were analyzed using Student’s unpaired *t* test to calculate statistical significance of observed differences. Test results with *p* values less than 0.05 were considered statistically significant (_*_, *p* < 0.05; _**_, *p* < 0.005; _***_, *p* < 0.001).

## 3 Results

### 3.1 MKP1 downregulates RBOHD activity through mechanisms independent of phosphosite targets of main RBOHD activating kinases

**Figure 1:**
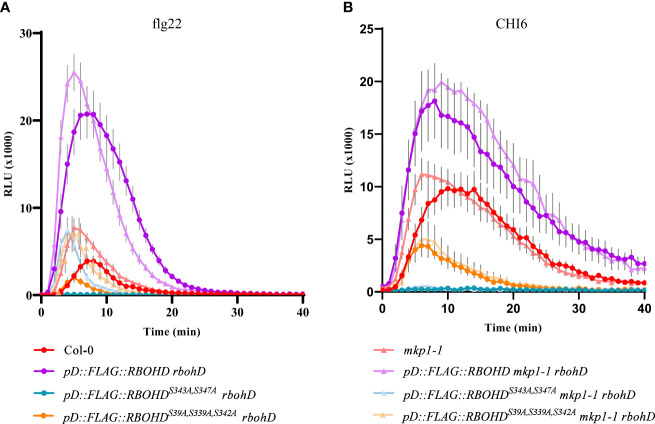
ROS production is increased in defective phosphosite RBOHD mutant alleles in *mkp1-1* background. H_2_O_2_ production after 1 μM flg22 **(A)** or 50 μM Chitohexaose-CHI6 **(B)** measured in a luminol-based assay using leaf discs from 4-week-old plants of the listed genotypes. *Arabidopsis thaliana* genotypes tested include Col-0 (WT), and *rbohD* lines complemented with *pD::FLAG::RBOHD, pD::FLAG::RBOHD^S39A/S339A/S343A^*, and *pD::FLAG::RBOHD^S343/S347^*, under both WT (*MKP1*) and *mkp1-1* mutant backgrounds. Relative light units (RLU) were measured over a period of 40 minutes. Values are average ± SE (n = 8). Data from one of three experiments performed that gave similar results. See **Supplementary Figure S1** for additional information.

MKP1 was shown to function as a negative regulator of MAMP-dependent ROS production, since *mkp1* mutant alleles (*mkp1-1* and *mkp1-2*) displayed enhanced ROS accumulation in response to flg22 and chitin (Escudero et al., 2019). Considering that induction of RBOHD, the key oxidase responsible of these ROS, is mainly achieved by its phosphorylation by different kinases (Castro et al., 2021; Wu et al., 2023), we hypothesized that MKP1 could antagonize with some of these regulatory kinases and dephosphorylate RBOHD to limit ROS production. To assess this hypothesis, we introduced in *mkp1-1 rbohD* and *rbohD* genetic backgrounds constructs harboring *RBOHD* wild-type (WT) gene fused to a tag (FLAG) under the control of its own promoter (*pD::FLAG-RBOHD* line), or *RBHOD* carrying mutations in the phosphosites of RBOHD that are the targets of activating kinases during immunity: Ser (S) to Ala (A) mutations in three phosphosites that are the target of BIK1 during PTI responses (*pD::FLAG::RBOHD^S39A/S339A/S343A^*; Kadota et al., 2014; Li et al., 2014) and in two phosphosites that are also activated in PTI and ETI responses (*pD::FLAG::RBOHD^S343A/S347A^*; Kadota et al., 2019). We monitored H_2_O_2_ production with these transgenic lines in response to diverse MAMPs. As described previously (Escudero et al., 2019), *mkp1-1* plants exhibited faster and enhanced H_2_O_2_ accumulation after bacterial MAMP flg22 treatment compared to WT Col-0 ecotype plants (**Figure 1A**). *rbohD* plants complemented with *pD::FLAG-RBOHD* exhibited higher ROS production than WT Col-0 plants upon flg22 treatment, that was not enhanced in *mkp1-1* background (**Figure 1A**). As described previously (Kadota et al., 2019), *rbohD* plants complemented with *pD::FLAG::RBOHD^S39A/S339A/S343A^* displayed reduced ROS production and *rbohD* complemented with *pD::FLAG::RBOHD^S343A/S347A^* almost abolished all H_2_O_2_ production after flg22 treatment. Interestingly, under *mkp1-1* background, ROS production was restored at *mkp1-1* levels in these two genotypes (*mkp1-1 rbohD pD::FLAG::RBOHD^S39A/S339A/S343A^* and *mkp1-1 rbohD pD::FLAG::RBOHD^S343A/S347A^*) in comparison to plants in *MKP1* WT background (**Figure 1A** and **Supplementary Figure S1A**). We also performed the same experiments after elicitation of all these genotypes with the fungal MAMP chitohexaose (CHI6), obtaining similar results, though the effect of *mkp1-1* mutation on ROS levels was lower in *pD::FLAG::RBOHD^S343A/S347A^* than in *pD::FLAG::RBOHD^S39A/S339A/S343A^* (**Figure 1B** and **Supplementary Figure S1B**). The fact that the *RBOHD* lines with altered phosphosites displayed enhanced ROS production under *mkp1-1* background compared to WT (*MKP1*) plants suggests that MKP1 could negatively regulate RBOHD by mediating dephosphorylation of some additional phosphosites to the ones that are targeted by BIK1 and the main regulatory kinases of RBOHD documented (Castro et al., 2021; Wu et al., 2023).

### 3.2 MKP1 does not directly interact and dephosphorylate RBOHD

**Figure 2:**
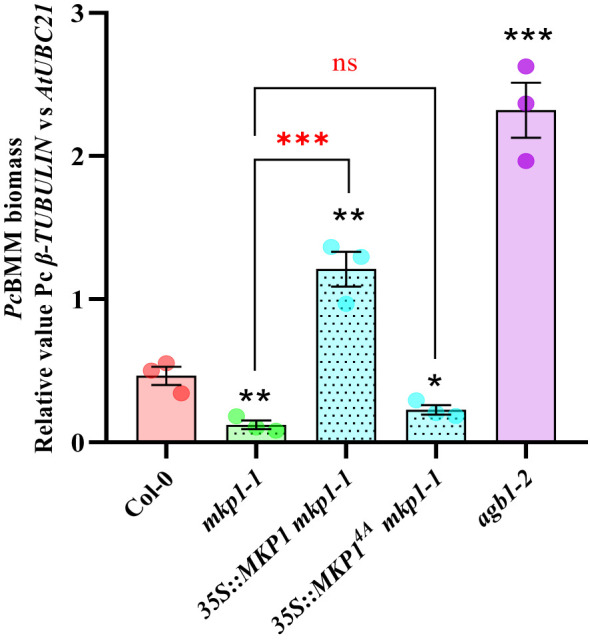
Phosphorylation sites of MKP1 are needed to complement *mkp1-1* defense phenotype. Seventeen-day-old plants of the listed genotypes were inoculated with a suspension of 4 x 10^6^ spores/ml of the fungus *P. cucumerina* (*Pc*BMM), and fungal biomass was quantified by qPCR at 5 days-post-inoculation (dpi). Quantitative PCR was performed with specific primers (*Pc*BMM *β-TUBULIN* and Arabidopsis *UBC21* genes) on gDNA extracted from inoculated plants (see Materials and Methods). Values are represented as average ± SE (n = 3) and are compared to Col-0 plants values. *agb1-2* was included in the experiments as a highly susceptible genotype for comparison. Horizontal keys compare the *mkp1-1* with the rest of the mutants. Black and red asterisks indicate values statistically different than Col-0 wild-type plants and *mkp1-1*, respectively according to Student’s t-test (*p < 0.05; **p < 0.01; ***p < 0.001; ns, no significant). Experiment was performed three times with similar results.

It was previously shown that expression of *35S::MYC::MKP1* construct in *mkp1-1* plants restored the susceptible phenotype against the bacterium *P. syringae* pv. tomato (*Pto*) DC3000 observed in WT plants (Jiang et al., 2017). Moreover, the use of *35S::MYC::MKP1^4A^ mkp1-1* plants, with the 4 putative regulatory phosphosites of MKP1 mutated to Ala (MKP1^4A^), showed that these modifications are essential to restore the susceptible phenotype against *Pto* DC3000, suggesting that MKP1 gets stabilized through phosphorylation following treatment with MAMPs (Jiang et al., 2017). To evaluate the extension of this requirement to other patho-systems, we examined the growth of the necrotrophic fungus *Pc*BMM on *mkp1-1* lines complemented with *35S::MYC::MKP1* and *35S::MYC::MKP1^4A^* (Jiang et al., 2017). Quantification of *Pc*BMM growth at 5 dpi revealed that *MKP1* overexpression lines (*35S::MYC::MKP1 mkp1-1)* exhibited increased enhanced fungal growth compared to the WT, further confirming MKP1 as a negative regulator of disease resistance (**Figure 2**). *MKP1* overexpression lines with mutated phosphosites (*35S::MYC::MKP1^4A^ mkp1-1)* displayed comparable resistance to *mkp1-1*, showing lower fungal growth than WT plants, and indicating that phosphorylation of MKP1 is required for its functional activity as negative modulator of the immune responses and disease resistance against the necrotrophic *Pc*BMM, as shown previously for *P. syringae* (Jiang et al., 2017).

**Figure 3:**
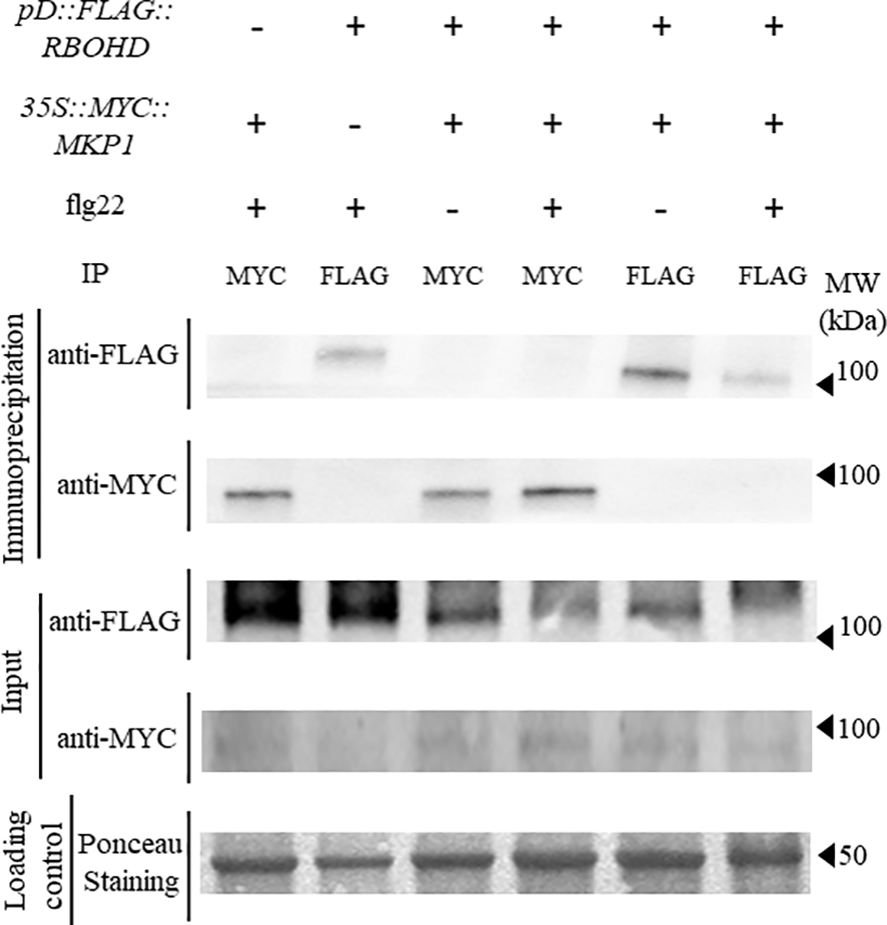
FLAG-RBOHD and MYC-MKP1 proteins do not interact in co-immunoprecipitation experiments. FLAG-RBOHD and MYC-MKP1 constructs were co-expressed in *Arabidopsis thaliana* by crossing *pD::FLAG::RBOHD* to *35S::MYC::MKP1 plants*, and selecting double homozygous plants in *rbohD mkp1-1* background. Total protein was extracted after elicitation of 12-day-old seedlings for 10 minutes with 500 nM flg22 (+) or H_2_O (-), and immunoprecipitated using monoclonal anti-FLAG or anti-MYC antibodies (on top of the gels). FLAG-RBOHD and MYC-MKP1 proteins were detected by western blot using anti-FLAG and anti-MYC antibodies in immunoprecipitated and control crude protein extracts (left of the gels). Loading control of crude extracts stained with red ponceau are shown to validate that the same concentration of protein was subjected to immunoprecipitation. Protein band corresponding to RUBISCO protein is shown in ponceau control. Molecular weight is indicated in kDa on the right. Experiment was performed twice with similar results.

Since we hypothesized that MKP1 could bind and dephosphorylate RBOHD to inactivate this oxidase, we crossed *35S::MYC::MKP1* plants to *pD::FLAG::RBOHD* and obtained double homozygous plants expressing both transgenes in double *mkp1-1 rbohD* mutant background to study putative interactions between MKP1 and RBOHD. We collected tissue from seedlings before and after elicitation with flg22, extracted proteins and performed co-immunoprecipitation studies. Immunodetection showed that anti-MYC antibody identified MYC-MKP1 only in samples immunoprecipitated with anti-MYC and independently of flg22/mock treatments. Conversely, anti-FLAG antibody only identified FLAG-RBOHD in samples immunoprecipitated with anti-FLAG (**Figure 3**). Therefore, lack of detection of FLAG-RBOHD and MYC-MKP1 in immunoprecipitated samples with anti-MYC and anti-FLAG, respectively, suggest that MKP1 and RBOHD do not interact directly, under the immunoprecipitation conditions tested. These experiments suggest that MKP1 would not directly mediate dephosphorylation and deactivation of RBOHD.

### 3.3 BIK1 and MKP1 mediate independent mechanisms of disease resistance

**Figure 4:**
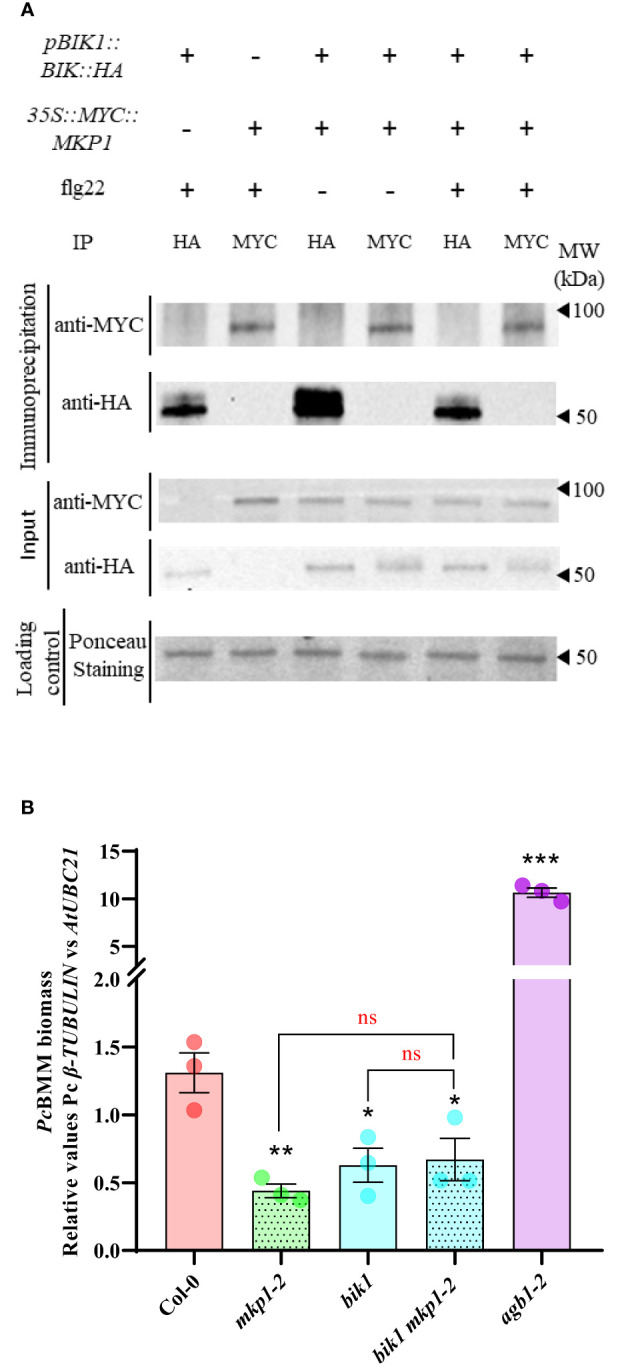
BIK1 and MKP1 mediate independent signaling pathways in immunity. **(A)** BIK1-HA and MYC-MKP1 did not interact in co-immunoprecipitation experiments. BIK1-HA and MYC-MKP1 construct were co-expressed in *Arabidopsis thaliana* by crossing *pBIK::BIK1::HA* to *35S::MYC::MKP1* plants, and total protein was extracted after elicitation for 10 minutes with 500 nM flg22 (+) or H_2_O(-), and immunoprecipitated using monoclonal anti-MYC or anti-HA antibodies (on top of the gels). MYC-MKP1 and BIK1-HA proteins were detected by western blot using anti-MYC and anti-HA antibodies in immunoprecipitated and control crude protein extracts (left of the gels). Loading control of crude extracts stained with red ponceau are shown to validate that the same concentration of proteins was subjected to immunoprecipitation. Protein band corresponding to RUBISCO protein is shown in ponceau control. Molecular weight is indicated in kDa on the right. Experiment was performed twice with similar results. **(B)** *bik1 mkp1-2* double mutant displays the same enhanced disease resistance to *P. cucumerina* BMM (*Pc*BMM*)* than single mutants. Seventeen-day-old plants of the listed genotypes were inoculated with a suspension of 4 x 10^6^ spores/ml of the fungus *Pc*BMM and fungal biomass was quantified by qPCR at 5 days-post-inoculation. Quantitative PCR was performed with specific primers (*Pc*BMM *β-TUBULIN* and Arabidopsis *UBC21* genes) on gDNA extracted from inoculated plants. *agb1-2* was included in the experiments as a highly susceptible genotype for comparison. Values represented are average ± SE (n = 3) and compared to Col-0 plants. Black asterisks above each mutant indicate values statistically different than Col-0 according to Student’s t-test (*, p < 0.05; **, p < 0.005; ***, p < 0.001). Horizontal keys compare the double *bik1 mkp1-2* with the individual mutants according to Student’s t-test (ns, no significant). The experiment was performed three times with similar results.

We therefore hypothesized that MKP1 would exert its negative regulation of RBOHD-dependent ROS production by targeting some of the kinases modulating activation of RBOHD. We focused our studies in BIK1, with a prominent role in the activation of RBOHD (Kadota et al., 2014; Li et al., 2014). BIK1 is activated through direct phosphorylation upon MAMP perception by PRR receptors, like FLS2 (receptor for flagellin peptide flg22; Gómez-Gómez et al., 1999) or EFR (receptor for elf18 peptide; Zipfel et al., 2006). Activated BIK1 triggers ROS production by phosphorylation of RBOHD residues Serine39, Serine339 and Serine343 (Kadota et al., 2014). Lack of BIK1 results in a significant reduction of ROS production after flg22 treatment (Li et al., 2014). We crossed *35S::MYC::MKP1* to *pBIK1::BIK1::HA* (Kadota et al., 2014), and performed protein extraction and co-immunoprecipitation studies in the F1 population before and after elicitation with flg22. Immunodetection showed that anti-MYC antibody identified MYC-MKP1 only in samples immunoprecipitated with anti-MYC and independently of the flg22 treatment. Similarly, anti-HA antibody only identified BIK1-HA protein in samples immunoprecipitated with anti-HA (**Figure 4A**). Therefore, these data indicate that MKP1 and BIK1 do not directly interact, under the immunoprecipitation condition tested. To further assess if there is a genetic interaction between *MKP1* and *BIK1*, we crossed the *bik1* mutant line to *mkp1-1* and *mkp1-2* and identified double mutants. *mkp1-1* harbors a T-DNA insertion in the Tyr-phosphatase dual specific domain and displays stronger abnormal growth than *mkp1-2*, which carries a W252 to stop codon mutation in the same domain (Escudero et al., 2019). Interestingly, both double mutants displayed enhanced abnormal phenotypes than the individual mutants, with the *bik1 mkp1-1* mutant exhibiting further heightened aberrant growth phenotype than *bik1 mkp1-2* (**Supplementary Figure S2**). We assessed pathogen resistance only in the double mutant *bik1 mkp1-2*, since the seeds obtained from *bik1 mkp1-1* were scarce. Both *bik1* and *mkp1-2* supported less *Pc*BMM growth than WT Col-0, and the double *bik1 mkp1-2* displayed a similar resistance phenotype than the individual mutants (**Figure 4B**). All these data suggest that BIK1 and MKP1 targets are different, and they modulate independent pathways.

### 3.4 MKP1 is a repressor of MPK3 signaling in response to a necrotrophic fungus

**Figure 5:**
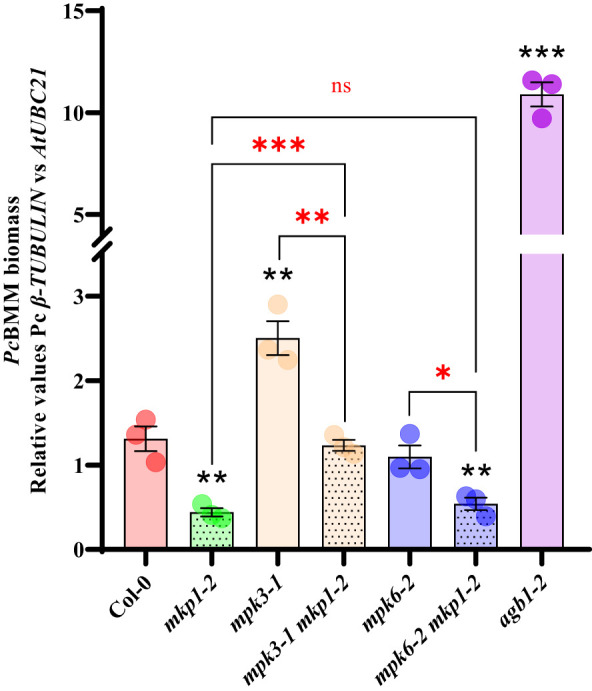
Enhanced disease resistance of *mkp1* to the necrotrophic fungus *P. cucumerina* is dependent of MPK3 function. *P. cucumerina* BMM (*Pc*BMM*)* biomass quantification in sixteen-day-old plants of the listed genotypes at 5 days-post-inoculation with a suspension of 4 x 10^6^ spores/ml of the fungus. Quantitative PCR was performed with specific primers (*Pc*BMM *β-TUBULIN* and Arabidopsis *UBC21* genes) on gDNA extracted from inoculated plants (see Material and Methods). *agb1-2* was included in the experiments as a highly susceptible genotype for comparison. Values represented are average ± SE (n = 3) and compared to Col-0 plants. Black asterisks above each mutant indicate values statistically different than Col-0 according to Student’s t-test (^*^ *p* < 0.05; ^**^ *p* < 0.005; ^***^ *p* < 0.001). Red marks above the keys indicate statistical differences according to Student’s t-test between genotypes (^*^, *p* < 0.05; ^**^, *p* < 0.005; ^***^, *p* < 0.001; ns, no significant). The experiment was performed three times with similar results.

To further characterize the defense signaling elements downregulated by MKP1, we generated combinatory mutants with *mkp1* lines and mutations affecting diverse elements regulating disease resistance downstream RBOHD and BIK1. Null mutations in *MPK3* and *MPK6* were shown to suppress some *mkp1* phenotypes. Developmental defects displayed by *mkp1-1* were partially suppressed in *mpk3 mkp1-1* and *mpk6 mkp1-1* double mutants (Bartels et al., 2009). Even though, *mpk3* and *mpk6* contributed differently to this suppression since each mutant was able to suppress different *mkp1-1* developmental defects (Bartels et al., 2009). Interestingly, the *mkp1-1* enhanced resistance to *P. syringae* required only *MPK6* (as *mpk6 mkp1-1* were less resistant than *mkp1-1*) but not *MPK3* (Bartels et al., 2009; Anderson et al., 2011). We wanted to expand these previous analyses with *P. syringae* to other pathogens with different colonization styles. Therefore, we generated double mutants *mpk3-1 mkp1-2* and *mkp6-2 mkp1-2* with a different *mkp1* allele and assessed the growth of the fungal pathogen *Pc*BMM on these lines. *mpk3-1* supported more fungal growth at 4 dpi than WT Col-0, whereas *Pc*BMM growth was unaltered in *mpk6-2* compared to Col-0 (**Figure 5**). The analysis of the double mutants revealed that *mpk3-1* mutation partially suppressed the *mkp1-2* resistance phenotype in *mpk3-1 mkp1-2*, whereas *mpk6-2* did not interfere with *mkp1-2* resistance since *mkp6-2 mkp1-2 *resistance to *Pc*BMM was similar to that of *mkp1-2* (**Figure 5**). Thus, contrary to what happens in the defensive response against *P. syringae*, enhanced *mkp1* disease resistance to *Pc*BMM seems to be partially dependent on *MPK3* but not on *MPK6*, suggesting some interaction between MKP1- and MPK3-mediated signaling pathways in response to *Pc*BMM infection.

### 3.5 MKP1 regulates distinct defensive pathways in response to pathogens with different lifestyles

**Figure 6:**
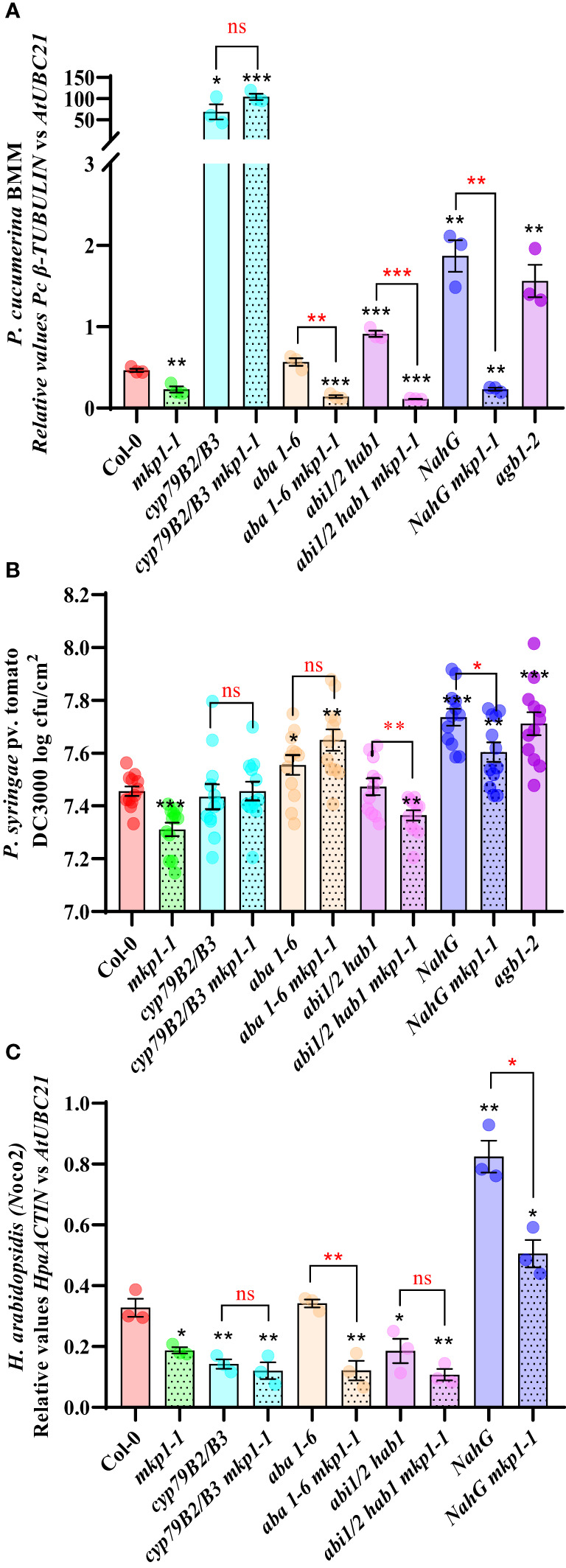
MKP1 regulates distinct defensive pathways in response to pathogens with different lifestyle. **(A)**. *P cucumerina* BMM *(Pc*BMM*)* biomass quantification in 17-day-old plants of the listed genotypes at 5 days-post-inoculation (dpi) with a suspension of 4 x 10^6^ spores/ml of the fungus. Quantitative PCR (qPCR) was performed with specific primers (*Pc*BMM *β-TUBULIN* and Arabidopsis *UBC21* genes) on gDNA extracted from the inoculated plants (see Material and Methods). Results are average ± SE (n = 3). **(B)**. Quantification of *P. syringae* pv. tomato DC3000 growth on 4-week-old plants of the indicated genotypes at 4 dpi after spray inoculation with a bacterial suspension (3 x 10^8^ colony forming units (cfu)/ml). Results are average cfu ± SE (n = 4). *agb1-2* was used as highly susceptible control in **(A, B)**. **(C)**. *H. arabidopsidis (Hpa)* Noco2 biomass quantification in 10-day-old seedlings of the listed genotypes at 6 dpi with 4 × 10^4^ conidiospores/ml. *Hpa* growth was determined by qPCR on gDNA extracted from the inoculated plants using oligonucleotides of *HpaACTIN* gene, and these values were normalized to *AtUBC. NahG* plants were used as highly susceptible control. Results in **(A–C)** are average ± SE (n = 3). Black asterisks indicate statistical significance levels according to Student´s *t* test (^*^, *p* < 0.05; ^**^, *p* < 0.01; ^***^, *p* < 0.001), compared to Col-0, wild-type plants. Red marks above black keys indicate statistical significance levels (Student´s *t* test) between genotype comparisons performed (^*^, *p* < 0.05; ^**^, *p* < 0.005; ^***^, *p* < 0.001; ns, no significant). These experiments were performed three times with similar results.

A metabolomic analysis performed on *mkp1-2* revealed the constitutive accumulation of metabolites related to SA signaling and Trp-derived secondary metabolites, and, in a lower extent, to elements related with abscisic acid (ABA) signaling (Escudero et al., 2019). To further decipher the molecular basis of *mkp1*-mediated resistance phenotypes, we generated combinatory mutants with the stronger mutant allele *mkp1-1* and lines disrupted in canonical signaling pathways potentially up-regulated in *mkp1-1* and required for disease resistance to different pathogens. We combined *mkp1-1* with: i) *cyp79B2 cyp79B3* double mutant (impaired in the Trp-derived secondary metabolites pathway needed for the biosynthesis of indol-glucosinolates like camalexin and indol-3-carboxylic acid; Bednarek et al., 2009); ii) *NahG* line (transgenic plant over-expressing a SA hydroxylase gene that degrades this hormone to catechol; Delaney et al., 1994); iii) *aba1-6* mutant (impaired in ABA biosynthesis; Niyogi et al., 1998); and *abi1 abi2 hab1* (abbreviated *abi1/2 hab1)* triple mutant that is hypersensitive to ABA since it is defective in negative regulators of ABA signaling, which have been described as ROS sensors (Bi et al., 2022). With these mutants we assessed the effect that alteration of different signaling pathways have on the disease resistance to pathogens with different lifestyle displayed by *mkp1-1* (**Figure 6**).

We first examined the growth of the necrotrophic fungus *Pc*BMM on these lines compared to Col-0 wild-type plants at 5 dpi (**Figure 6A**). We used *agb1-2*, a mutant defective in the heterotrimeric G-protein β-subunit, as hypersusceptible control (Escudero et al., 2019). Whereas *NahG* and *abi1/2 hab1* lines were slightly more susceptible than the control, *cyp79B2 cyp79B3* mutant was extremely susceptible to this pathogen compared to Col-0, as described previously (Sanchez-Vallet et al., 2010). Interestingly, *cyp79B2 cyp79B3 mkp1-1* displayed the same enhanced susceptibility than *cyp79B2 cyp79B3*, whereas the combination of *mkp1-1* with lines altered in SA signaling *(NahG)* or ABA signaling (*aba1-6* mutant and *abi1/2 hab1)* did not interfere with *mkp1-1* resistance (**Figure 6A**). These data indicates that depletion of the Trp-derived metabolites pathway in *cyp79B cyp79B3* suppresses the enhanced resistance displayed by *mkp1-1* to this necrotrophic fungus, whereas mutations in the other pathways have minor or no effect on *mkp1-1* mediated resistance. Therefore, MKP1 seem to negatively regulate the Trp-derived metabolites pathway in response to this necrotrophic pathogen, since *mkp1-1* plants constitutively accumulate Trp-derived secondary metabolites (Escudero et al., 2019) and *mkp1-1* resistance to *Pc*BMM is lost in *cyp79B2 cyp79B3* background.

We also performed similar resistance analyses in response to the hemibiotrophic bacterium *Pto* DC3000 with the generated lines (**Figure 6B**). *agb1-2* mutant was also used as hypersusceptible control in these experiments (Delaney et al., 1994; Torres et al., 2013). *NahG* and *aba1-6* lines were slightly more susceptible at 4 dpi than Col-0, whereas *cyp79B2 cyp79B3* supported comparable bacterial growth than the control Col-0 plants. Interestingly, *cyp79B2 cyp79B3*, *aba1-6* and *NahG* combinations with *mkp1-1* abolished *mkp1-1* resistance (**Figure 6B**). In contrast, in *mkp1-1 abi1/2 hab1* quadruple mutant the level of resistance to *Pto* DC3000 was similar to that of *mkp1-1* plants (**Figure 6B**). These data indicate that both SA and ABA signaling and Trp-derived metabolites contribute to the disease resistance phenotype to this pathogen observed in *mkp1-1* plants, suggesting that MKP1 negatively regulates all three pathways in response to *Pto* DC3000.

We then assessed growth of the biotrophic oomycete *Hpa* isolate Noco2 in these genotypes (**Figure 6C**). In this interaction *NahG* plants were the hypersusceptible control (Delaney et al., 1994). Among the different lines affected in the three signaling pathways evaluated only *NahG* plants showed enhanced susceptibility to this oomycete compared to Col-0, as described previously (Rairdan and Delaney, 2002). *NahG* was able to suppress the resistance phenotype displayed by *mkp1-1*, whereas all the rest of *mkp1-1* combinations did not impede its enhanced resistance (**Figure 6C**). Notably, we found that c*yp79B2 cyp79B3* supported lower *Hpa* growth than the control Col-0 plants, and that *mkp1-1* c*yp79B2 cyp79B3* lines showed a similar level of resistance to that of *mkp1-1.* Moreover, the *aba1-6* line showed no alterations in its level of disease resistance whereas the *abi1/2 hab1* lines displayed a slight lower growth of *Hpa* in comparison to Col-0, but these mutations did not suppress the enhanced resistance of *mkp1-1* to *Hpa* (**Figure 6C**). These data indicate that, in response to this pathogen, MKP1 seem to mainly negatively regulate SA signaling, as downregulation of this pathway suppresses *mkp1-1* resistance to *Hpa*. Also, our data point that, in addition to the previously described contribution of SA to disease resistance to this biotrophic oomycete (Lawton et al., 1995; Rairdan and Delaney, 2002), some metabolites synthesized through CYP79B2 CYP79B3 might have some negative effect on *Hpa* disease resistance (**Figure 6C**).

**Figure 7:**
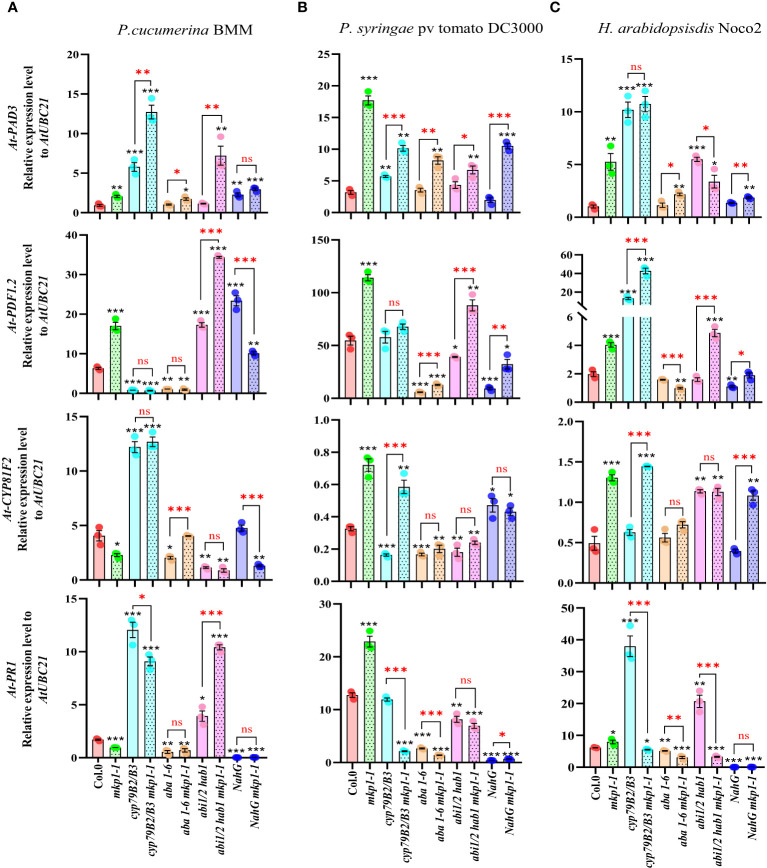
Expression analyses of defense marker genes in genotypes harboring *mkp1-1* allele upon infection with pathogens with different lifestyle. qRT-PCR analyses of expression of defense marker genes in the indicated genotypes at 3 days-post-inoculation (dpi) with pathogens: **(A)** *P. cucumerina* BMM, performed on 17-day-old plants; **(B)** *P. syringae* pv. tomato DC3000, performed on 4-week-old plants; and **(C)** *H. arabidopsidis* Noco2, performed on 11-day-old seedlings. Expression levels of *PAD3*, *PDF1-2, CYP81F2* and *PR1* genes were quantified relative to housekeeping gene *UBC21.* Data represented are average ± SE of three technical replicates from 3 experimental replicates. Black asterisks indicate statistical significance levels compared to Col-0 wild type plants, according to Student´s *t* test (^*^, *p* < 0.05; ^**^, *p* < 0.005; ^***^, *p* < 0.001). Red asterisks above black keys indicate the statistical significance levels (Student´s *t* test) between genotypes comparisons performed (^*^, *p* < 0.05; ^**^, *p* < 0.005; ^***^, *p* < 0.001; ns, no significant). These experiments were performed three times with similar results.

To further assess the signaling elements regulated by MKP1 in response to these pathogens with different lifestyles and to establish a connection between the enhanced resistance phenotypes conferred by *mkp1-1* mutation in different genetic backgrounds to the expression of immune-related genes, we monitored the transcription of various marker genes at 3 dpi with the three pathogens (**Figure 7**). We monitored the expression of: i) *PR1*, marker of SA signaling (Uknes et al., 1992); ii) *PDF1-2*, plant defensin, marker of jasmonic acid (JA)/ethylene (ET) signaling (Penninckx et al., 1996); iii) *PAD3* (encoding an enzyme of the Trp-derived metabolites pathway that catalyzes the last step of camalexin biosynthesis: Zhou et al., 1999); and iv) *CYP81F2* (encoding a monooxygenase of the Trp-derived metabolites pathway that mediates the production of some indole-glucosinolates; Bednarek et al., 2009). We found that *mkp1-1* plants showed higher expression levels of *PDF1-2* and *PAD3* than Col-0 plants upon infection with the three pathogens, and that the expression of *PR1* and *CYP81F2* genes was also higher in *mkp1-1* than in Col-0 upon *Pto* and *Hpa* infection (**Figure 7**). The expression of these genes in non-inoculated *mkp1-1* and Col-0 plants, and the rest of genotypes tested, was quite similar (**Supplementary Figure S3**), indicating that these genes exhibited enhanced up-regulated upon infection in *mkp1-1* plants in comparison to Col-0, further corroborating the negative function of MKP1 in the control of disease resistance responses. The analysis of the expression of these genes in different combinatorial genetic lines and in response to infection with the three pathogens tested revealed a great complexity of transcriptional responses controlled by MKP1, and identified some patterns of expression that might explain the increased resistance of some genotypes harboring *mkp1-1* mutation. For example, upon infection with *Pc*BMM, the expression of *PAD3, PDF1-2* and *PR1*, but not *CYP81F2*, was enhanced in *abi1/2 hab1 mkp1-1* in comparison to *abi1/2 hab1*, and the expression of *PAD3* and *CYP81F2* was higher in *aba1-6 mkp1-1* than in *aba1-6* (**Figure 7A**). These patterns of expression could explain the enhanced resistance of these two lines harboring *mkp1-1* mutation. In contrasts, the expression of these four genes was not enhanced in *NahG mkp1-1* in comparison to *NahG* plants upon *PcBMM* infection (**Figure 7A**), indicating that the observed reduced susceptibility of *NahG mkp1-1* in comparison to *NahG* plants (**Figure 6A**) was not associated to a regulatory effect of MKP1 on the pathways triggering the expression of these genes. In the infection with *Pto*, *mkp1-1* mutation just reduced the susceptibility of *NahG* plants to the bacterium in *NahG mkp1-1* line (**Figure 6B**) that showed an enhanced expression of *PAD3* and *CYP81F2*, suggesting that regulation of MKP1 on Trp-derived metabolites pathway might explain *NahG mkp1-1* reduced susceptibility phenotype (**Figure 7B**). In the infection of plants with *Hpa*, the reduction of susceptibility to this pathogen in *NahG mkp1-1* and *aba1-6 mkp1-1* in comparison to *NahG* and *aba1-6* plants (**Figure 6C**) could just be associated to a slight increased expression of *PAD3* in lines harboring *mkp1-1* mutations (**Figure 7C**). Together these expression analyses revealed the complexity of the interactions between MKP1 and some immune pathways, which are shown here to also depend on the pathogen tested.

## 4 Discussion

The activation of disease resistance requires a delicate balance between ensuring an effective defense against invading pathogens and maintaining plant development processes, that should not be compromised to guarantee plant fitness and offspring (Huot et al., 2014; Monson et al., 2022). Central to this balancing process is the regulation of ROS production, a key component of plant immune responses, but that also can be potentially harmful molecules and cause cell death in the plant (Mittler, 2017). In *Arabidopsis thaliana*, MKP1 has emerged as a crucial negative regulator that limits ROS production and long-lasting plant defense responses, having an impact on the control of broad-spectrum disease resistance mechanisms (Bartels et al., 2009; Escudero et al., 2019; Anderson et al., 2011; Jiang et al., 2017). The presented study delves into the molecular intricacies of MKP1-mediated immune regulation, shedding light on its involvement in diverse immune pathways.

### 4.1 Negative regulation of RBOHD-dependent ROS production by MKP1 is not achieved by their direct interaction

A pivotal aspect explored in this study is the negative regulation of ROS production mediated by MKP1. The NADPH oxidase RBOHD is the primary contributor to pathogen-induced ROS in *Arabidopsis thaliana* (Torres et al., 2002). *mkp1* mutant alleles (*mkp1-1* and *mkp1-2*) produce faster and higher level of RBOHD-dependent H_2_O_2_ accumulation than WT plants in response to MAMPs (**Figure 1**; Escudero et al., 2019). We and others (Jiang et al., 2018) hypothesized that MKP1 could directly dephosphorylate RBOHD and contribute to deactivate its oxidase activity to prevent excessive ROS production or to make the ROS burst transient. Several residues of RBOHD, particularly Ser343 and Ser347 in the N-terminus, are the convergent point for several activating kinases (Wu et al., 2023). Interestingly, the use of *rbohD* complemented lines with *RBOHD* alleles carrying mutations in these residues (S343A/S347A) that are the target of activating kinases during ETI and PTI, as well as in the serines targeted by the central immune regulator BIK1 (S39A/S339A/S343A), revealed that these mutated versions of RBOHD protein produced more ROS in *mkp1-1* than in WT (*MKP1*) background (**Figure 1**). This is indicative that the putative MKP1 dephosphorylation targets in RBOHD would be additional residues than the ones targeted by the main regulatory kinases. However, our co-immunoprecipitation studies did not reveal a direct interaction between MYC-MKP1 and FLAG-RBOHD, even though the respective proteins were distinctly detected (**Figure 3**). We cannot rule out that other methodologies to determine protein/protein interaction *in vivo* (Bracha-Drori et al., 2004) would show interaction between RBOHD and MKP1 proteins, but we estimate that MKP1 does not achieve this regulation of ROS production through a direct interaction with RBOHD.

### 4.2 BIK1 and MKP1 mediate independent pathways

As an alternative, we theorized that MKP1 would target and downregulate some of the kinases that activate RBOHD in immunity. We focused our study on BIK1, an essential player in this activation under an important control mechanism (Kadota et al., 2014; Li et al., 2014). In the resting state, within the PRR complex, BIK1 undergoes ubiquitination and is subsequently directed for proteasomal degradation. Upon MAMP recognition, the PRR complex phosphorylates and releases BIK1 being further stabilized/activated by SIK1 kinase that also directly targets RBOHD (Zhang et al., 2018). However, co-immunoprecipitation experiments with MYC-MKP1 and BIK-HA did not show direct interaction between these two proteins (**Figure 4A**), making unlikely that MKP1 deactivates BIK1 to negative regulate RBOHD-dependent ROS production. The results obtained with plants harboring the allele *RBOHD^S39A/S339A/S343A^* with mutations in the main BIK1 targets (**Figure 1**) also pointed partially to this conclusion, since MKP1 contribution to ROS level produced by this allele is scarce in *mpk1-1 rbohD pD::FLAG::RBOHD^S39A/S339A/S343A^* plants. Despite the acknowledged roles of both MKP1 and BIK1 in immune regulation, the analysis of *bik1 mkp1* double mutants reveals non-epistatic interactions in relation to the developmental phenotypes mediated by these genes (**Supplementary Figure S2**), indicating distinct targets for these proteins. This independence raises intriguing questions about the redundancy and specificity within the plant immune system, suggesting that MKP1 and BIK1 mediate diverse signaling pathways in response to different pathogens. Even though both *mkp1-2* and *bik1* exhibit enhanced resistance to *Pc*BMM compared to Col-0 control, and the resistance phenotype of the double mutant is not additive and cannot be differentiated from the individual mutants (**Figure 4B**). These results might indicate some epistatic interaction at the level of disease resistance regulation between *MKP1* and *BIK1*. It might be also possible that the method used to determine the level of enhanced resistance to *Pc*BMM does not discriminate between subtle resistance differences since *mkp1-2* and *bik1* showed already a significant reduction of fungal growth.

Alternatively, MKP1 could exerts this negative function on ROS production acting through the repression of other PTI components such as MPK regulation. MKP1 has been shown to interact with MPK3/MPK6, among other MPKs (Ulm et al., 2002; Jiang et al., 2018), and to repress these two MPKs in stress-related signaling (Bartels et al., 2009; Anderson et al., 2011) and in cell fate decision during stomatal development (Tamnanloo et al., 2018). However, the use of a chemical-genetic conditional loss-of-function *mpk3 mpk6* double mutant demonstrated that the flg22-triggered ROS burst is independent of MPK3/MPK6 activation (Xu et al., 2014), making unlikely that MKP1 repression of RBOHD-dependent ROS production is produced through these MPKs. Interestingly, we found that MKP1 mode of action involve some differential mechanism of regulation on MPK3 and MKP6 during disease resistance. In response to *P. syringae* MPK1 appears to repress specifically MPK6, as *mpk6 mkp1-1* were less resistant than *mkp1-1* (Bartels et al., 2009; Anderson et al., 2011). However, in the analysis of the resistance response to the necrotrophic fungus *P. cucumerina BMM* determined here, MKP1 seems to repress specifically MPK3, as *mpk3-1 mkp1-2* plants were less resistant than *mkp1-2* to the fungus (**Figure 5**). Therefore, MKP1 would target distinct MPKs in response to plant colonization by diverse pathogens, further indicating that MKP1 is able to differentially regulate distinct defensive pathways downstream PRRs in response to different pathogens.

### 4.3 MKP1 controls the orchestration of distinct defensive pathways in response to pathogens with different lifestyles

To broaden our understanding of how MKP1 negatively regulates immunity, we examined its impact on defensive pathways involved in disease resistance against pathogens with diverse lifestyles. Analysis of mutant combinations, involving alterations in the synthesis of Trp-derived secondary metabolites, SA and ABA signaling pathways, revealed the selective regulation exerted by MKP1 (**Figure 6**). This study presents compelling evidence that MKP1’s influence on disease resistance against three different pathogens varies in distinct mutant combinations, underscoring its ability to differentially regulate defensive pathways depending on the nature of the invading pathogen. Indeed, in response to the necrotrophic *P. cucumerina* BMM, where Trp-derived secondary metabolites play a significant role in controlling the pathogen progression (Pastorczyk et al., 2020; Bednarek et al., 2009; Sanchez-Vallet et al., 2010), MKP1 appears to predominantly negatively regulate this pathway (**Figure 6A**). Conversely, in the case of the interaction with the biotrophic oomycete *H. arabidopsidis* Noco2, where SA signaling plays a major role (Rairdan and Delaney, 2002), MKP1 is shown to primarily regulate this signaling pathway (**Figure 6C**). Furthermore, when responding to the hemibiotrophic bacterium *P. syringae* DC3000, where various signaling pathways contribute to its immunity (Rairdan and Delaney, 2002; De Torres-Zabala et al., 2007), MKP1 downregulates all three evaluated defensive mechanisms: Trp-derived metabolites, SA and ABA signaling (**Figure 6B**). The results showed here suggest that MKP1 negative modulating function in *Arabidopsis thaliana* disease resistance is quite complex and depends on the pathogen infecting the plant. Of note, a recent study by Lin et al. (2022) reveals that *Arabidopsis thaliana mkp1* plants exhibit increased susceptibility to vascular-adapted bacterial pathogens such as *Xanthomonas campestris* pv. campestris and display compromised nonhost resistance against the rice pathogen *Xanthomonas oryzae* pv. oryzae. This susceptible phenotype is explained by the demonstration that MKP1 underlies a tissue-specific mechanism by positively regulating lignin biosynthesis, that appears to be a crucial aspect of vascular-specific immunity (Jiang et al., 2022; Lin et al., 2022: Molina et al., 2021).

Our analysis of defense marker gene expression further corroborates the differential *MKP1* regulation of defense signaling depending on the pathogen. The upregulation of *PAD3* and *CYP81F2* in response to *P. cucumerina* BMM, and the fact that the later gene is further upregulated in *mkp1-1* background, confirm the importance of Trp-derived metabolites in the interaction with this necrotrophic pathogen (Piślewska-Bednarek et al., 2018; Pastorczyk et al., 2020; Sanchez-Vallet et al., 2010) (**Figure 7A**). The elevated level of SA-regulated *PR1* expression also shown in this interaction in the *cyp79B2 cyp79B3* double mutant would be rather related to the high increase in pathogen growth supported by this mutant background (**Figure 6A**), since this marker gene is also induced during pathogen infection with virulent isolates (Uknes et al., 1992; Rogers and Ausubel, 1997). In line with this hypothesis, *mkp1-1 cyp79B2 cyp79B3* plants showed lower *PR1* expression than *cyp79B2 cyp79B3* background in response to these three pathogens (**Figure 7**) accordingly to their reduced level of infection in comparison to *cyp79B2 cyp79B3* mutants (**Figure 6**).

Several hormones contribute uniquely to the plant’s ability to mount an effective defense against pathogens with diverse lifestyles. An antagonistic relationship between the SA and JA/ET pathways have been documented to mediate defense strategies based on the nature of the pathogen (Pieterse et al., 2012). SA plays a central role in inducing resistance against biotrophic pathogens by activating defense-related genes (Durrant and Dong, 2004). Conversely, JA and ET are primarily associated with defense against necrotrophic pathogens (Lorenzo et al., 2003). In contrast to SA, JA and ET, ABA is generally considered a negative regulator of plant defense, interfering with the activation of the main signaling hormones mediating defenses against these different pathogens (Adie et al., 2007). Even though ABA can enhance plant resistance against certain pathogens by promoting stomatal closure, thereby restricting pathogen entry (Cao et al., 2011). Our resistance analysis together with the defense gene expression studies point to a preeminent role of MKP1 in regulating the production of Trp-derived secondary metabolites that would in turn regulate other signaling elements. In fact, auxins are metabolites derived from Trp that, besides having some effects in disease resistance, also promote susceptibility by antagonizing with other hormones (Navarro et al., 2006; Robert-Seilaniantz et al., 2011). Thus, MKP1 primary regulation of Trp-derived metabolites could interfere with other hormone signaling contributing to broaden the effect mediated by MKP1 on immunity.

### 4.4 Conclusion

In conclusion, this study reveals the multifaceted role of MKP1 in orchestrating diverse immune responses in *Arabidopsis thaliana* against pathogens with different lifestyles. Beyond just understanding MKP1’s role in immunity, we also learn how it negatively regulates ROS production and various signaling pathways, providing a basis for strengthening our understanding of plant defenses mechanisms. Manipulating the expression or activity of MKP1 might be a way to generate crop protection against different pathogens. Moreover, deciphering the specificity of MKP1 in modulating different defensive pathways might open avenues for generating crops varieties (e.g. using genome editing technologies) with broad spectrum disease tailored to specific agricultural environments. However, a better understanding of the impact of inactivating MKP1 function on plant fitness would be essential to design enhanced resistance crops that do not show detrimental effect on yield. Notably, various crop species (e.g. tomato) contain several orthologs of MKP1 in their genomes and therefore it might be possible to increase crops broad spectrum disease resistance without compromising yield by impairing just one of MKP1 orthologs. Indeed, a recent article shows that mutations in *MKP1* in wheat produce plants that are not only more resistant to two devastating fungal pathogens but also exhibit a higher yield compared to wild-type control plants without infection (Liu et al., 2024).

## Data availability statement

The original contributions presented in the study are included in the article/**Supplementary Material**. Further inquiries can be directed to the corresponding authors.

## Author contributions

MT: Conceptualization, Formal analysis, Investigation, Methodology, Project administration, Supervision, Writing – original draft, Writing – review & editing. DB: Formal analysis, Investigation, Methodology, Writing – original draft. AM: Conceptualization, Funding acquisition, Project administration, Resources, Supervision, Writing – original draft, Writing – review & editing.
