# Supplementary material for: Mitogen-activated protein kinase phosphatase 1 controls broad spectrum disease resistance in Arabidopsis thaliana through diverse mechanisms of immune activation
Source: Front Plant Sci. 2024 Mar 21;15:1374194. doi: 10.3389/fpls.2024.1374194 (PMC10993396; doi:10.3389/fpls.2024.1374194)
Supplement: Supplementary file 1 [file Image_1.pdf]

Supplementary Figure S1

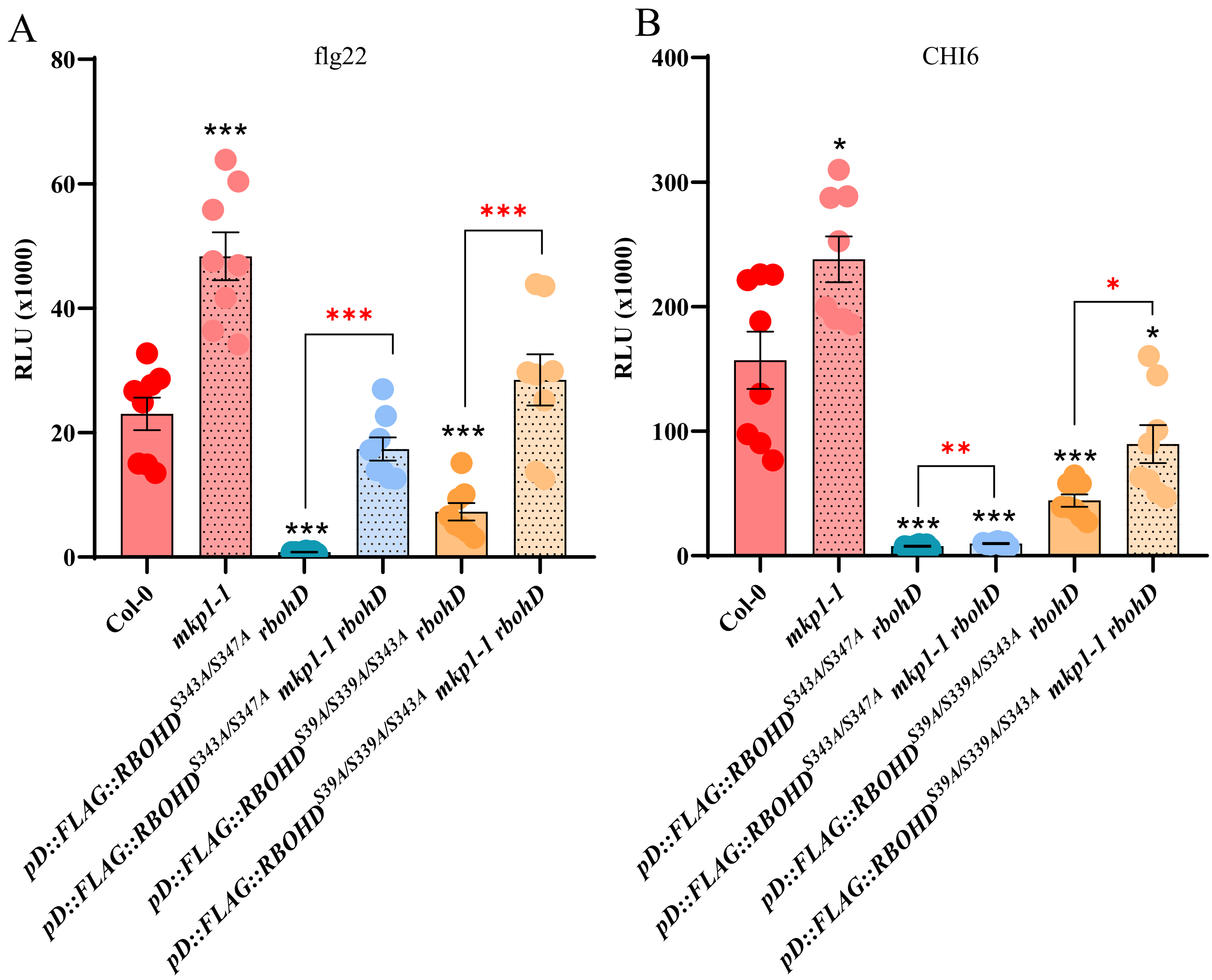

SUPPLEMENTARY FIGURE S1 | Quantification of the increased ROS production in defective phosphosite RBOHD mutant alleles in *mkp1-1* background. Total sum of H_2O_2 production after treatment with 1 µM flg22 **(A)** or 50 µM Chitohexaose-CHI6 **(B)** measured in a luminol-based assay using leaf discs from 4-week-old plants of the listed genotypes. Data include a selection of lines from **Figure 1**: Col-0 (WT), *mkp1-1* and *rbohD* lines complemented with, *pD::FLAG::RBOHD*$^{S39A/S339A/S343A}$ and *pD::FLAG::RBOHD*$^{S343/S347}$, under both WT (*MKP1*) and *mkp1-1* mutant backgrounds. Sum of relative light units (RLU) were measured over a period of 40 minutes. Values are average of total fluorescence for each genotype ± SE (n = 8). Black asterisks indicate statistical significance levels according to Student´s *t* test (*, *p* < 0.05; **, *p* < 0.005; ***, *p* < 0.001), compared to WT plants. Red asterisks above the keys indicate statistical differences between genotypes in WT or *mkp1* background according to Student's t-test (*, p < 0.05; **, p < 0.005; ***, p < 0.001; ns, no significant). Data from one of three experiments performed, all with similar results.
