# Supplementary material for: Mitogen-activated protein kinase phosphatase 1 controls broad spectrum disease resistance in Arabidopsis thaliana through diverse mechanisms of immune activation
Source: Front Plant Sci. 2024 Mar 21;15:1374194. doi: 10.3389/fpls.2024.1374194 (PMC10993396; doi:10.3389/fpls.2024.1374194)
Supplement: Supplementary file 2 [file Image_2.pdf]

Supplementary Figure S2

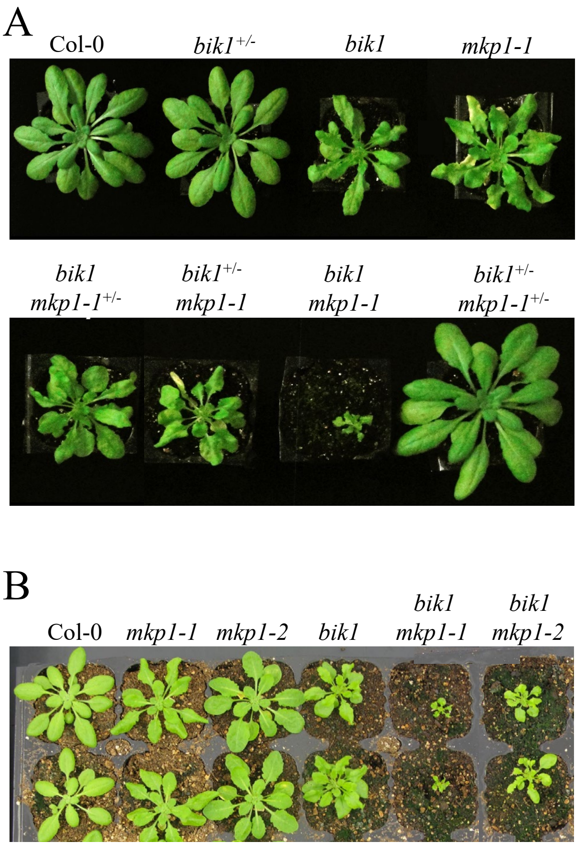

SUPPLEMENTARY FIGURE S2 | Double mutant *bik1 mkp1-1* displays enhanced aberrant phenotype than *bik1 mkp1-2* (A) *bik1 mkp1-1* double mutant displays an enhanced aberrant growth in comparison to *bik1* and *mkp1-1* single mutants. Pictures were taken at 25 days-post-gemination on plants grown under short day conditions (see Methods and Materials). (B) Image of 4-week-old plants of the listed genotypes grown under short day conditions (see Methods and Materials).
