# Supplementary material for: Mitogen-activated protein kinase phosphatase 1 controls broad spectrum disease resistance in Arabidopsis thaliana through diverse mechanisms of immune activation
Source: Front Plant Sci. 2024 Mar 21;15:1374194. doi: 10.3389/fpls.2024.1374194 (PMC10993396; doi:10.3389/fpls.2024.1374194)
Supplement: Supplementary file 3 [file Image_3.pdf]

Supplementary Figure S3

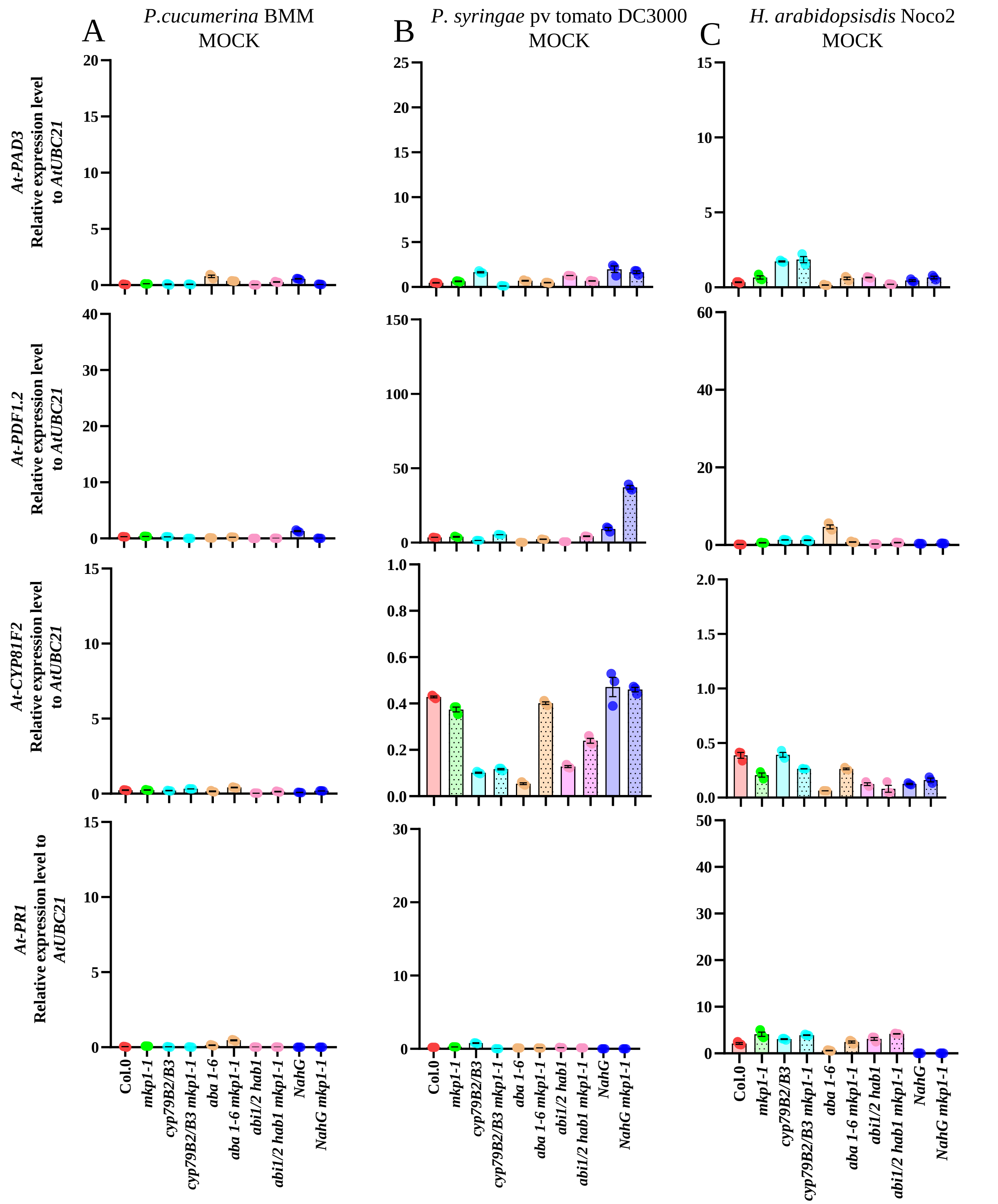

SUPPLEMENTARY FIGURE S3 | Expression analyses of defense marker genes in non-infected genotypes harboring *mkp1-1* allele. qRT-PCR analyses of expression of defense marker genes in the indicated genotypes measured 3 days after mock (water) treatment in parallel to the experiment shown in **Figure 7**. **(A)** 17-day-old plants; **(B)** 4-week-old plants; **(C)** 11-day-old seedlings. Expression levels of *PAD3*, *PDF1-2*, *CYP81F2* and *PR1* genes were quantified relative to housekeeping gene *UBC21*. Data represented are average ± SE of three technical replicates from 3 experimental replicates. Significant differences in the expression of the genes were not detected. These experiments were performed three times with similar results.
